# Metastatic mesenchymal chondrosarcoma showing a sustained response to cabozantinib: A case report

**DOI:** 10.3389/fonc.2022.1086677

**Published:** 2022-12-12

**Authors:** Veronika Blum, Vanghelita Andrei, Baptiste Ameline, Silvia Hofer, Bruno Fuchs, Klaus Strobel, Anna Allemann, Beata Bode, Daniel Baumhoer

**Affiliations:** ^1^ Oncology Department, Luzerner Kantonsspital, Luzerne, Switzerland; ^2^ Bone Tumour Reference Center, Institute of Medical Genetics and Pathology, University Hospital Basel, Basel, Switzerland; ^3^ Neurology Department, University Hospital and University of Zurich, Zurich, Switzerland; ^4^ Pathology Institute Enge, University of Zurich, Zurich, Switzerland

**Keywords:** mesenchymal chondrosarcoma, metastatic disease, p16 loss, tyrosine kinase inhibitors, case report, cabozantinib

## Abstract

Mesenchymal chondrosarcoma is a rare and aggressive sarcoma subtype with high risk for distant metastases and poor prognosis. Currently NCCN- and ESMO-Guidelines recommend using Ewing sarcoma protocols as standard treatment. Nevertheless, in localized disease overall 5-year survival rates are below 50% whereas in metastatic spread median progression-free survival rates of only 5 months can be expected. Here we present a patient with metastatic osseous spread of mesenchymal chondrosarcoma that showed a sustained clinical improvement and a good partial response on imaging over a period of one year when treated with the multi-tyrosine kinase inhibitor cabozantinib. Although we cannot explain the exact mechanism underlying this treatment effect, tumors with similar genetic patterns might respond to the same therapy as well.

## Introduction

Mesenchymal chondrosarcoma (MCS) is a rare sarcoma subtype accounting for less than 5% of all chondrosarcomas. The *HEY1::NCOA2* gene fusion is present in virtually all MCS and is considered the main driver of tumorigenesis through different mechanisms including direct DNA-binding, protein-protein-interaction and epigenetic modification (1, 2). Recently, it was shown that the main downstream targets of *HEY1::NCOA2* are *PDGFRA*, *PDGFRB* and *BCL2 (* 3). Optimal treatment for patients with localized MCS remains controversial. Due to an aggressive behavior and high risk for distant metastases, complete resection together with (neo-)adjuvant treatment according to Ewing sarcoma (ES) protocols is recommended according to the NCCN- and ESMO-Guidelines (4). Nonetheless, the prognosis for MCS remains poor with 5-year survival rates not exceeding 40-50% (5, 6). In metastatic disease, response rates to chemotherapy are generally below 30% and median progression-free survival rates of five months have been reported (7).

Cabozantinib is a broad acting multi-kinase-inhibitor which targets MET, VEGFR-1, VEGFR-2, VEGFR-3, KIT, NTRK2, FLT3, AXL, RET, TEK, ROS1, TYRO3, and MER. Efficacy of cabozantinib has recently been described in recurrent ES and osteosarcoma as well as in selected soft tissue sarcomas (8–11). To the best of our knowledge, no patients with metastatic MCS treated with cabozantinib have been reported so far.

## Case report

**Figure 1:**
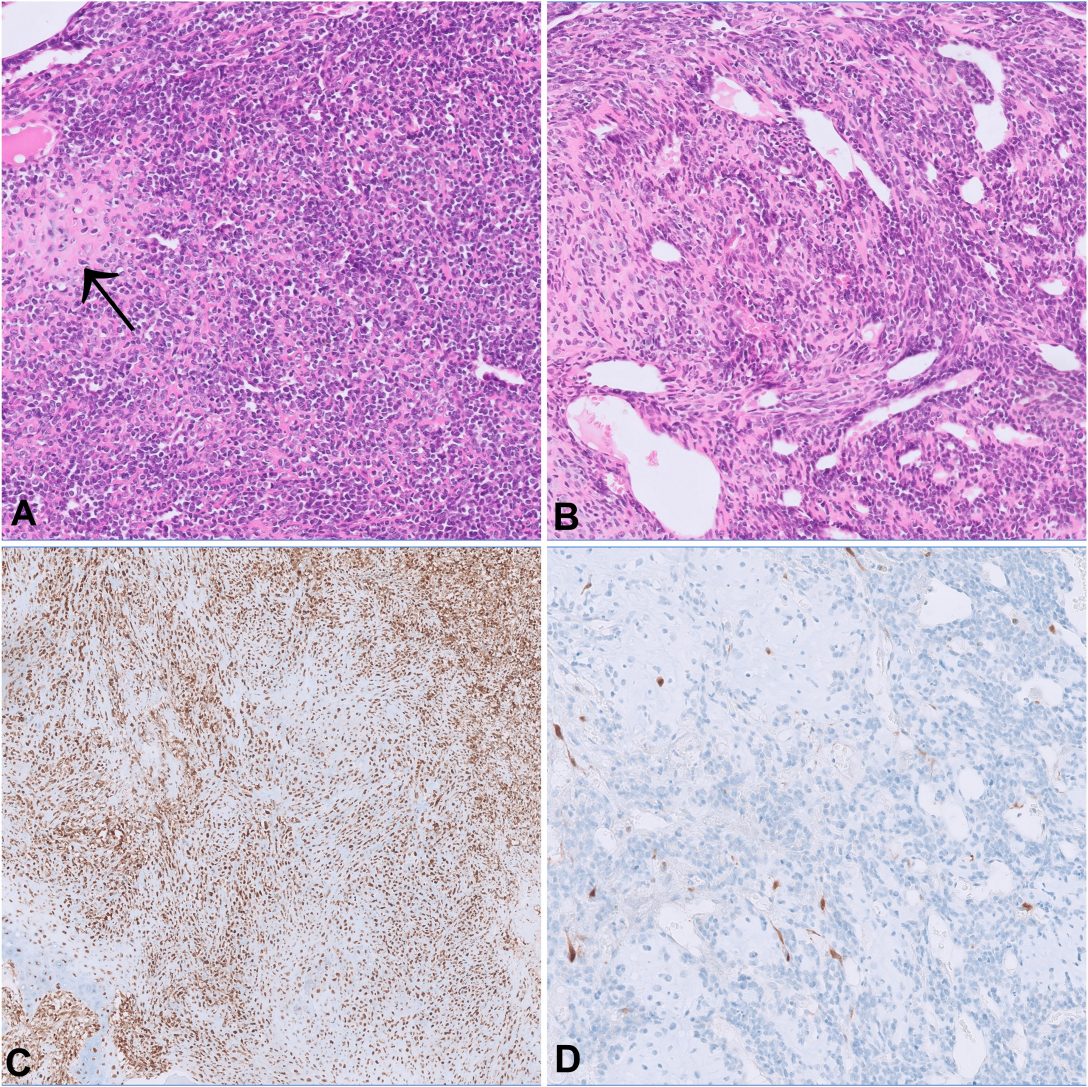
The morphology of both the primary tumor **(A)** and the metastasis from the femoral head **(B)** show cellular sheets of small round to spindle cells merging with foci of cartilaginous differentiation (arrow). On immunohistochemistry, p16 expression is retained in the primary tumor **(C)** and lost in the metastasis **(D)**.

A 47-year-old female with no relevant medical history was diagnosed with localized MCS of the right tibia harboring the disease defining *HEY1::NCOA2* fusion. The biopsy showed a biphasic tumor consisting of solid sheets of small round to spindle cells admixed with islands of hyaline cartilage (**Figure 1**). The patient declined neoadjuvant chemotherapy and a resection with clear margins was performed. One and a half years later, she presented with pain in her lower spine. A FDG-PET-CT scan revealed multiple osseous lesions in the spine, pelvis, both proximal femurs, and several ribs, which were confirmed to represent metastatic disease on biopsy.

**Figure 2:**
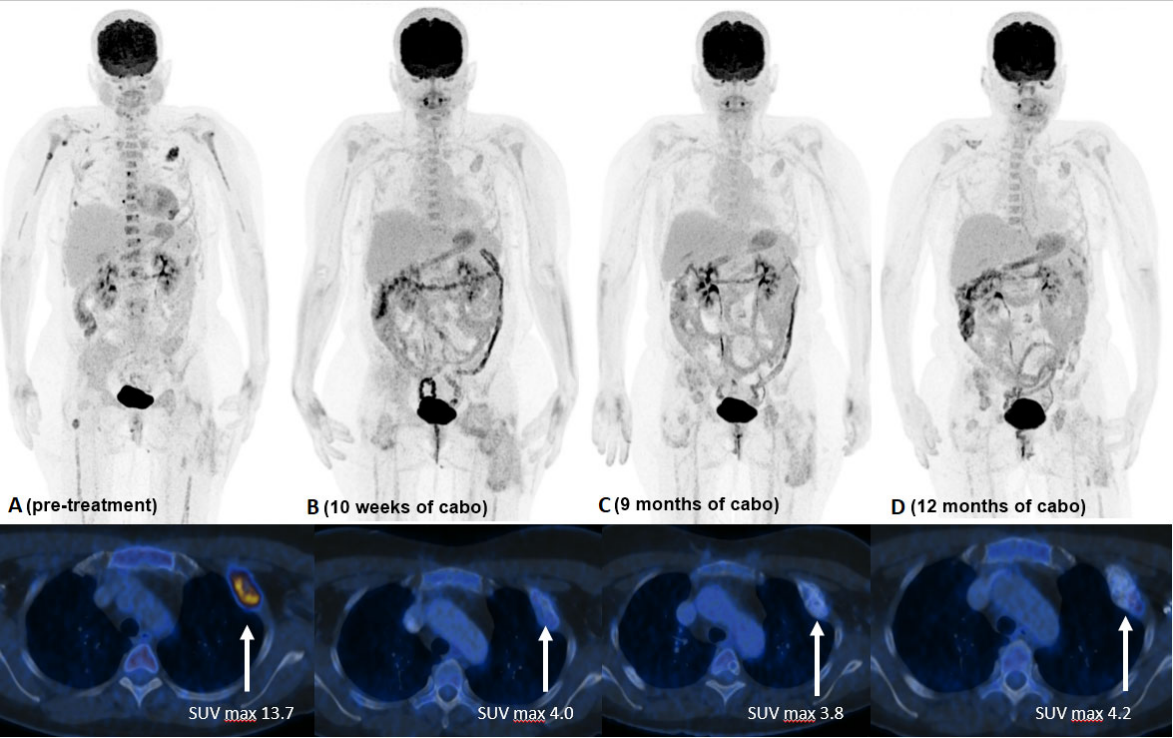
FDG PET/CT [**(A)** 7/21] with multiple FDG avid bone metastases (ribs, spine, femurs, humerus, skullcap). SUV max, in the large left sided rib metastasis 13.7. FDG PET/CT [**(B)** 10/21] 10 weeks after start of TKI treatment with reduction of uptake in all bone metastases (rib lesion reduction to SUV max. 4.0). No new FDG avid lesions visible. FDG PET/CT [**(C)** 4/22] 9 months and [**(D)** 7/22] 12 months after TKI start with ongoing good response.

Due to myelocompression and a high risk of fracture, the metastasis in the third lumbar vertebrae was treated by debulking and spondylodesis from T12 to S1. The left proximal femur was also prophylactically stabilized by osteosynthesis after curetting the metastasis. Postoperative radiotherapy was performed in the region of L3-5, both hips and the right ilium, each with 13 x 3 Gy. Subsequently, the irradiated areas showed no FDG-uptake in the PET-CT anymore, but new and partly progressive osteolytic lesions on the left skullcap, spine, ribs, both femurs, and humerus were detected (**Figure 2A**).

**Figure 3:**
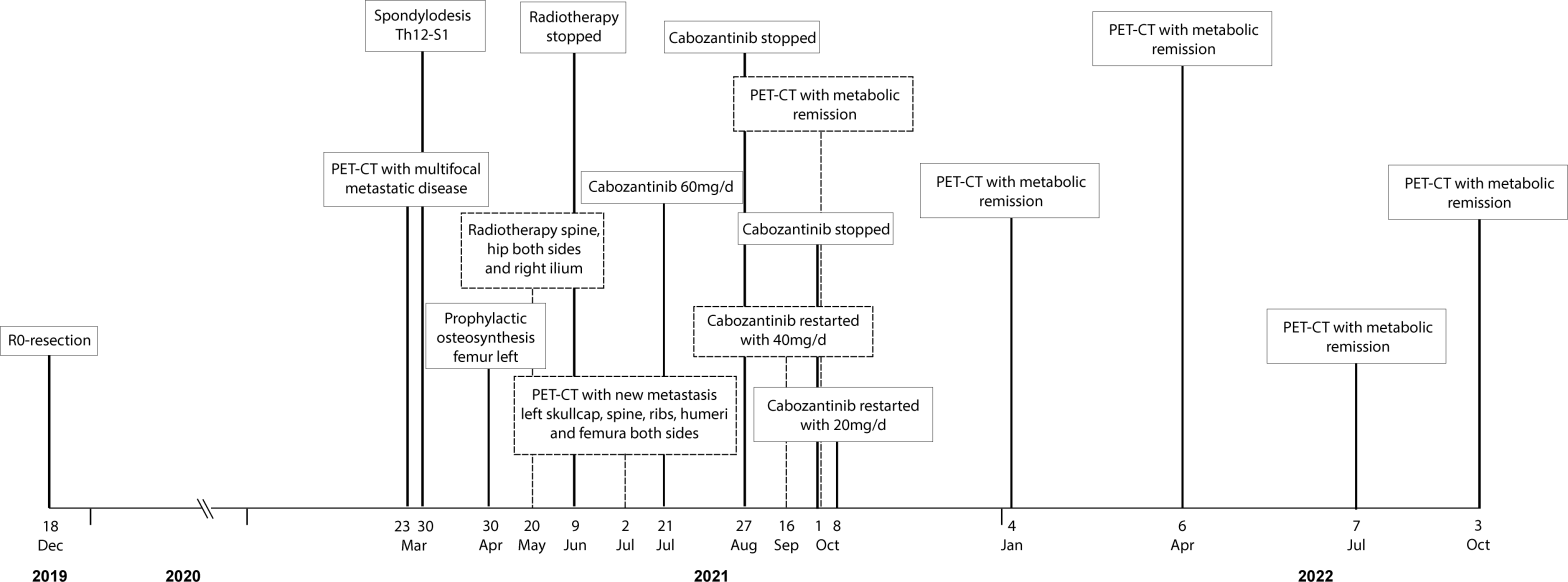
Detailed timeline showcasing relevant data from the episode of care.

Despite severe bone pain, the patient still refused to undergo conventional chemotherapy. Encouraged by the recently published data on the efficacy of cabozantinib in patients with recurrent ES and osteosarcoma (9), the drug was offered as an alternative approach, which she agreed to. Cabozantinib was started at a dosage of 60mg, administered once daily. Bone pain increased during the first two weeks of treatment, but steadily improved thereafter. Due to a palmar-plantar erythrodysesthesia, dosage had to be reduced to 40mg/d after the first cycle and to 20mg/d after 6 weeks of treatment. The patient tolerated Cabozantinib 20 mg/d without further adverse events. A FDG PET-CT scan performed 10 weeks after the beginning of treatment showed a partial metabolic response in all metastasis: as an example SUV max of a rib metastasis decreased from 13.7 to 4.0 and no new manifestations developed. Additional FDG PET-CT scans carried out after 27, 37, 48, and 60 weeks confirmed a sustained partial response (**Figure 2**). A detailed timeline with relevant data from the episode of care is presented in **Figure 3**. Clinically, the patient described a significant reduction of pain requiring only occasional oral analgesics, improvement in general condition and mobility.

## Materials and methods

Formalin-fixed paraffin-embedded tissue samples of the primary tumor and metastases from three different sites (lumbar spine, femur and ilium) were subjected to copy number and methylome analysis. Copy number variations were inferred from the Infinium Human Methylation Epic Array platform using the R-package conumee after pre-processing of raw data using the R-package minfi as described before (12). A tissue sample from the primary tumor was additionally investigated by Oncomine Comprehensive Panel version 3 and Archer FusionPlex Custom V2 Panel (Thermo Fisher Scientific, USA) according to routine protocols.

## Results

**Figure 4:**
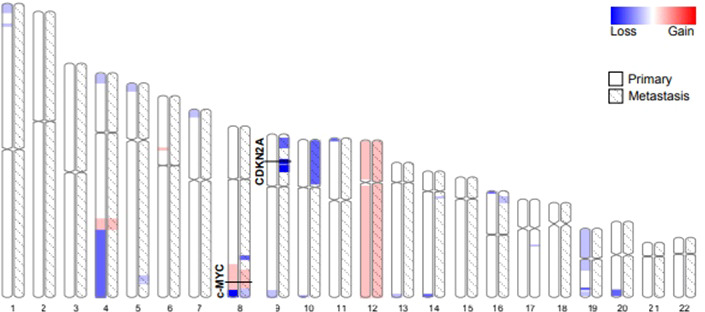
Autosome ideograms comparing copy number variations of the primary tumor (blank) and a metastasis (hatched). Both copy number profiles have been derived from DNA methylation arrays. The magnitude of gains (red) and losses (blue) are represented by a color gradient. The two loci specifically addressed in this study (*c-Myc* and *CDKN2A*) are highlighted.

Both the primary tumor and the metastases histologically showed a consistent biphasic pattern of undifferentiated small round to spindle cells with intermingled islands of well-differentiated hyaline cartilage, typical for MSC (**Figure 1**). The tumor defining *HEY1-NCOA2* gene fusion was identified in the primary tumor using the Archer FusionPlex Custom V2 Panel (Thermo Fisher Scientific, USA). DNA methylation profiling revealed high similarity with the established methylation class MSC using unsupervised clustering approaches following dimension reduction (data not shown) (13, 14). The copy number profiles of both the primary tumor and all three metastases showed aneuploidy of chromosome 12 as well as copy number variations of well-established cancer drivers (**Figure 4**). Among them, a low-level copy number gain of *MYC* was noted, which was confirmed by FISH analysis in a subset of tumor cells, supporting the interpretation of a subclonal event. Of note, all metastases showed a homozygous loss at 9p21.3 harboring *CDKN2A* (p16), that was absent in the primary tumor. This finding was confirmed by immunohistochemistry, with consistent and strong positivity of p16 in the primary tumor and loss of expression in the metastases (**Figure 1**). All three metastases shared identical DNA methylation and copy number profiles suggesting that they developed from an identical clone. An Oncomine Comprehensive Panel v3 (Thermo Fisher Scientific, USA) was performed in one of the metastases and did not reveal any point mutations within 135 cancer genes (including *RB1*).

## Discussion

The use of TKI in patients with sarcoma in advanced stages not responding to conventional treatment strategies are currently being intensively studied. The use of levantinib, a different TKI, is currently under evaluation in the treatment of selected sarcomas including osteosarcoma and chondrosarcoma (NCT04784247). This drug acts through the inhibition of VEGFR and impairs the activity of tyrosine kinase receptors, such as RET, similar to cabozantinib (15). Moreover, recent findings highlight the efficacy of Lenvatinib alone or in combination with denosumab compared to denosumab alone in treating bone sarcoma cell models, which is in line with the involvement of VEGFR in osteolytic lesions (16, 17). Additionally, several case reports of chondrosarcoma presenting prolonged disease control when treated with the TKI pazopanib were reported (18, 19).

Predictive biomarkers to identify patients that might benefit from these innovative approaches are urgently needed. Potential therapeutic targets have been described in various sequencing studies but usually using retrospective case series in which the efficacy of a targeted approach can no longer be validated (20, 21). Here, we present a patient with widespread metastatic MCS that showed an impressive and long lasting response to cabozantinib. The primary tumor as well as three metastases were comprehensively investigated for molecular alterations.

In a subset of tumor cells, we found a low level amplification of *MYC*. Despite being a well known stimulator of *VEGFA*, which is a direct target of cabozantinib, subclonal *MYC* amplification is unlikely to have caused the clinical response to treatment. As a strong inhibitor of *MET*, cabozantinib might have acted through downstream targets of the MCS-related chimeric protein itself. Indeed, *HEY1* is a mediator and effector of the activated Notch developmental and stemness pathway (1) and crosstalks between Notch and *MET* signaling have been described (22). Recently it has furthermore been shown that the fusion protein also activates *PDGFRA* and *PDGFRB* and dramatically increases phospho-AKT, thus stimulating the PDGF-PI3K-AKT axis (3) which is another target of cabozantinib.

In contrast to the primary tumor, the bone metastases showed a consistent and homozygous loss of the tumor suppressor gene *CDKN2A* (*p16*). Loss of *p16* results in increased Cyclin D1 and CDK4/6 activity promoting the partial phosphorylation of the C-terminal region of *RB1*. As a consequence, pRB1 can release E2F transcription factors which initiate the mitotic G1-S transition. This cascade is known to stimulate additional pathways, such as PDGF-PI3K-AKT and RAS-RAF-ERK MAPK, thereby promoting uncontrolled cellular proliferation, neoangiogenesis and tumor microenvironment remodeling. Loss of *p16* has been associated with tumor progression and a poor prognosis in various tumors, including MCS (20). However, a similar dramatic response to the kinase inhibitor Palbociclib has been reported in a 62-year-old patient with advanced chordoma which was also deficient of *p16 *(21).

Increased cellular proliferation induces hypoxia and thereby triggers a shift to anaerobic metabolism in the neoplastic cells through the HIF-1 pathway which stimulates VEGF expression and neoangiogenesis. Additionally, in a hypoxic state, activated VEGF can lead to imbalances in the ANG-TEK system, which further dysregulates the angiogenic signaling network. The TEK ligands Angiopoietin 1 and 2 promote angiogenesis in the presence of VEGF-A, whereas TEK-expressing macrophages support neovascularization by recruiting endothelial cells, and also promote a dissemination of tumor cells. Inhibiting the tyrosine kinase activity of TEK, MET, RET, FLT3, NTRK2, and AXL, cabozantinib blocks these disrupted angiogenic pathways. Moreover, targeting the tumor microenvironment through VEGF and TEK receptors directly downregulates tumor-induced angiogenesis which sustains tumor cell migration, extracellular matrix invasion and tumor related macrophages recruitment.

The activity of cabozantinib in the patient presented here is therefore likely explained by inhibition of a complex interplay between different signaling cascades, addressing both downstream targets of the *HEY1::NCOA2* fusion, as well as tumor microenvironment. The limitation of this study is our inability to elucidate the exact pathway underlying the response to the treatment with only one case.

## Conclusion

Finding the optimal treatment for patients with rare sarcomas is challenging, particularly because systematic clinical studies are usually not feasible. Our case illustrates an unexpected sustained clinical response to the multi-kinase-inhibitor Cabozantinib in a patient with metastatic mesenchymal chondrosarcoma with comprehensive molecular work-up. Although we cannot explain the exact mechanism underlying this treatment effect, this being the most important limitation of this study, tumors with similar genetic patterns might respond to the same therapy as well. Identifying genetic patterns requires more tumors to undergo systematic sequencing that should include copy number alterations as panel sequencing of 135 cancer genes did not reveal any pathogenic mutations in the case presented here. Taken together, our case illustrates that even in advanced sarcomas, targeted treatment approaches can show impressive clinical responses that should whenever possible be correlated with sequencing data.

## Data availability statement

The original contributions presented in the study are included in the article/supplementary material, further inquiries can be directed to the corresponding author.

## Ethics statement

The studies involving human participants were reviewed and approved by Ethikkommission beider Basel (reference 274/12). The patients/participants provided their written informed consent to participate in this study.

## Author contributions

DB conceived and designed the study. BA analysed and interpreted the methylation data. DB, VB, and VA prepared the manuscript with contributions from all other authors. All authors contributed to the article and approved the submitted version.
